# Hemodynamic Parameters Predict In-stent Thrombosis After Multibranched Endovascular Repair of Complex Abdominal Aortic Aneurysms: A Retrospective Study of Branched Stent-Graft Thrombosis

**DOI:** 10.3389/fcvm.2021.654412

**Published:** 2021-04-23

**Authors:** Ming-Yuan Liu, Yang Jiao, Junjun Liu, Simeng Zhang, Wei Li

**Affiliations:** ^1^Department of Vascular Surgery, Beijing Friendship Hospital, Capital Medical University, Beijing, China; ^2^Beijing Center for Vascular Surgery, Beijing, China; ^3^Department of Vascular Surgery, Peking University People's Hospital, Beijing, China; ^4^The Key Laboratory of Molecular Cardiovascular Science, Ministry of Education, Beijing, China; ^5^Department of Vascular Surgery, The Affiliated Hospital of Qingdao University, Qingdao, China; ^6^Department of Vascular Surgery, Changhai Hospital, Shanghai, China; ^7^Department of Pediatric Cardiac Surgery, State Key Laboratory of Cardiovascular Disease, Fuwai Hospital, National Center for Cardiovascular Disease, Chinese Academy of Medical Sciences and Peking Union Medical College, Beijing, China

**Keywords:** in-stent thrombosis, branched stent-grafts, computational fluid dynamic, endovascular aortic repair (EVAR), biomechanic

## Abstract

**Background:** Branch vessel occlusion is reported in endovascular repair of aortic pathology. This study aimed to evaluate the hemodynamic indicators associated with in-stent thrombosis (IST) of a branched stent-graft (BSG) after endovascular aortic repair (EVAR) of a complex abdominal aortic aneurysm.

**Methods:** A retrospective evaluation was performed based on the computed tomography (CT) scans and clinical data of three patients who underwent multi-branched endovascular repair. Patient-specific 3-dimensional models were reconstructed, and hemodynamic analysis was performed for IST. Hemodynamics-related parameters including time-averaged wall shear stress (TAWSS), oscillatory shear stress index (OSI), and relative residence time (RRT) were compared among the individual patients.

**Results:** The flow velocity, TAWSS, OSI, and RRT were radically changed in the area of the IST. In BSGs, IST tended to occur in the regions of hemodynamic alteration near the bends in the device, where a decreased flow velocity (<0.6 m/s) and TAWSS (<0.8 Pa) and an elevated OSI (>0.2) and RRT (>5 s) were consistently observed.

**Conclusions:** Hemodynamic perturbations in BSGs cause a predisposition to IST, which can be predicted by a series of changes in the flow parameters. Early hemodynamic analysis might be useful for identifying and remediating IST after multibranched endovascular repair.

## Introduction

The endovascular aortic repair (EVAR) has emerged as the “first choice” to treat abdominal aortic aneurysm (AAA) (1). For patients with thoracoabdominal or para-renal aneurysms that that not anatomically amenable to standard EVAR (2), a variety of approaches (3–5), including the physician modified stent grafts (PMSGs), the custom-made devices (CMDs) and the off the shelf devices, have been reported. However, the branched stent-graft (BSG) occlusion caused by the in-stent thrombosis (IST) has been reported in the clinical studies (6, 7), often leading to organ malperfusion (8). Morphological analysis alone might be insufficient to comprehensively evaluate the IST. Hemodynamics is considered to play a critical role in the formation and progression of IST (9, 10). Hemodynamic parameters might reveal the flow status of a BSG and provide quantifiable and even predictable evaluations of the IST, thus helping to inform medical decision making. However, the mechanism and hemodynamic characteristics of IST in BSG are poorly understood. Therefore, we aimed to evaluate the IST-related hemodynamic features of stent-grafts in this study.

## Methods

### Patient Data

**Figure 1:**
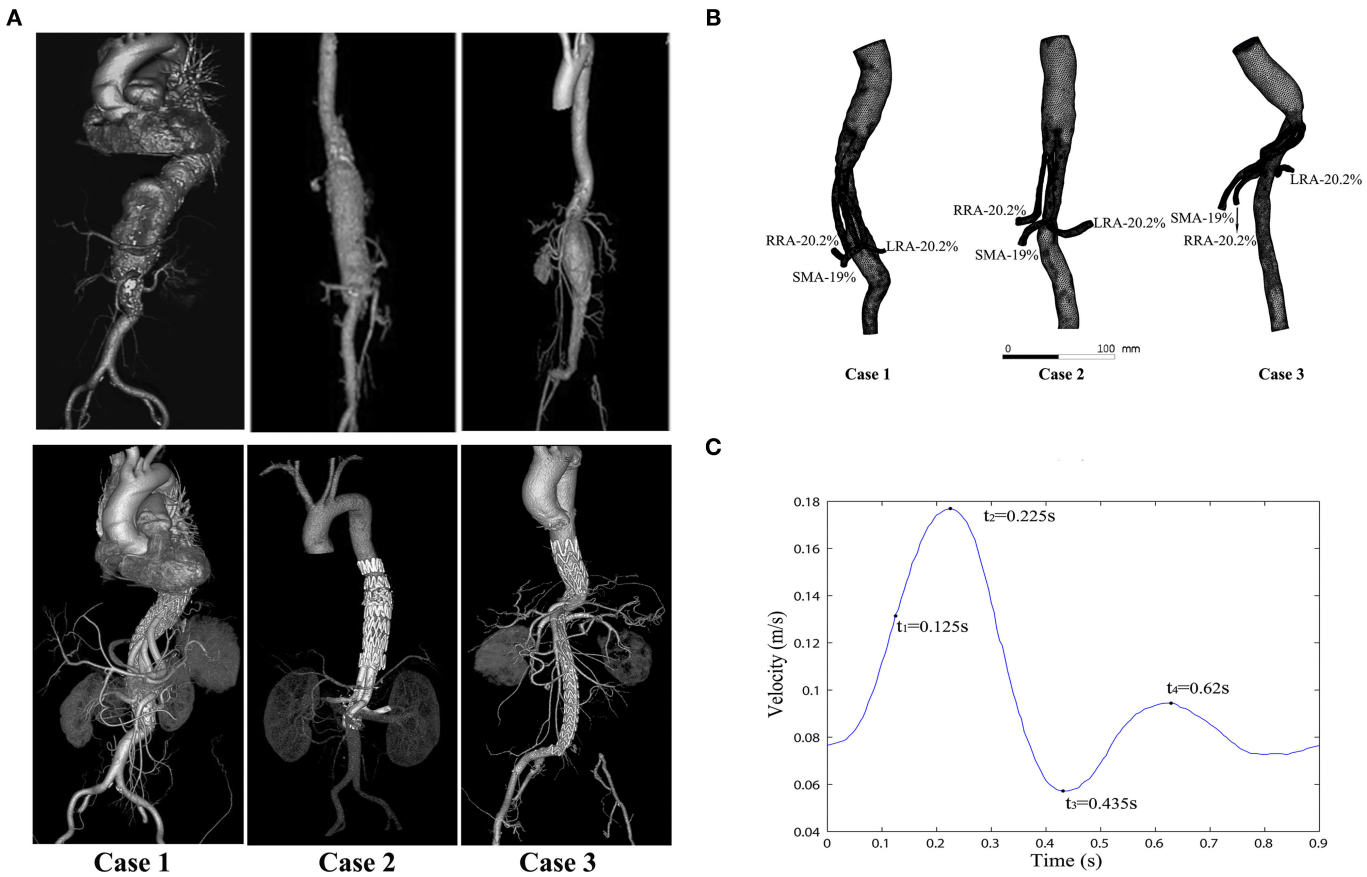
Computed tomography (CT) images and modeling images. **(A)** The CT angiograms (pre- and postoperative) of the three cases; the preoperative CT angiograms of these cases are shown in the top row, and the postoperative CT angiograms of the same cases are shown in the bottom row. **(B)** Meshes of the three models and the flow split ratio of each single branch vessel in the three cases. **(C)** Inlet velocity waveform of the aorta (four typical moments are marked in a cycle: *t*_1_ = 0.125 s, *t*_2_ = 0.225 s (the peak systolic moment), *t*_3_ = 0.435 s (the end systolic moment), and *t*_4_ = 0.62 s).

**Figure 2:**
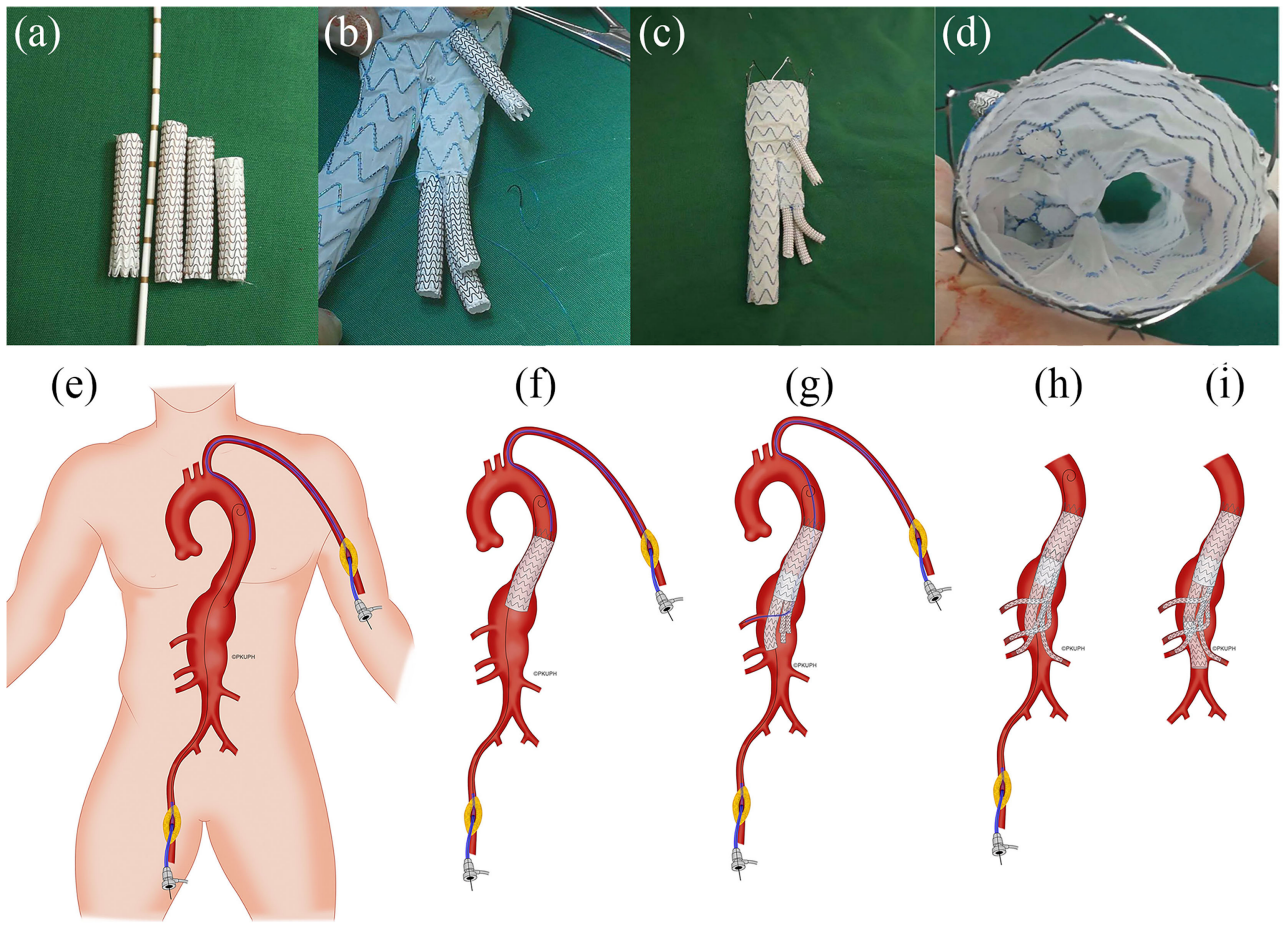
Representative illustration of the construction of physician-modified branched stent-grafts and the process of stent-graft implantation. **(a)** Splitting a Gore Viabahn^TM^ into 3 sections. **(b)** Sewing the caudally directed cuff to a Medtronic Endurant^TM^ bifurcated stent-graft. **(c)** Completion of the modification. **(d)** Top view of the visceral cuff showing no gutters. **(e)** The operation was performed using right femoral and left brachial access. **(f)** A Valiant Captivia^TM^ thoracic stent-graft was first deployed at the descending aorta. **(g)** Implantation of the main body of the modified Endurant bifurcated stent-graft through the right femoral approach and cannulation of the celiac arteries preferentially through left axillary artery access using a long sheath and an extra-stiff guidewire. **(h)** Sequential bridging of the visceral arteries with Viabahn while reinforcing the bilateral renal arteries using a self-expandable bare metal stent dilated from the target vessel to the cuff. **(i)** The distal iliac limb extension was docked, and flow was restored to the lower limbs (Department of Vascular Surgery, Peking University People's Hospital; all rights reserved).

The CT images of three patients were imported into Mimics software (v19.0, Materialize, Ann Arbor, MI, USA) to build three-dimensional (3D) models. All CT scan images were obtained from Peking University People's Hospital (Beijing, China). Informed consent was obtained from all the patients, and the study protocols were approved by the Ethical Review Board and the Statistics Department of Peking University People's Hospital (No. 2017 PHB166-01). The pre- and postoperative CT scan images of three cases are shown in the upper and lower rows of Figure 1A, respectively. The mesh models of the three cases (based on the CT scan images) were all obtained at the 1 month follow-up and shown in Figure 1B. All three of these patients with complex AAAs were treated with physician-modified stent grafts (PMSGs) repair for the aorta (Endurant™, Medtronic, Santa Rosa, Calif, USA) and the visceral arteries (Viabahn™, W. L. Gore, Flagstaff, AZ, USA). The patients were administrated with anti-platelet (aspirin 100 mg/per day) and anticoagulants (Rivaroxaban 10 mg/per day) during the first month, and only administrated with anti-platelet (aspirin 100 mg/per day) thereafter. The procedural details are illustrated in Figure 2. The orientation of the proximal part of the branched stent grafts was modified based on the measurement of the preoperative CTA. The orientation of the cuff branches was preoperatively analyzed, and the stent-grafts were deployed according to the preoperative measured angle and clock bit.

**Figure 3:**
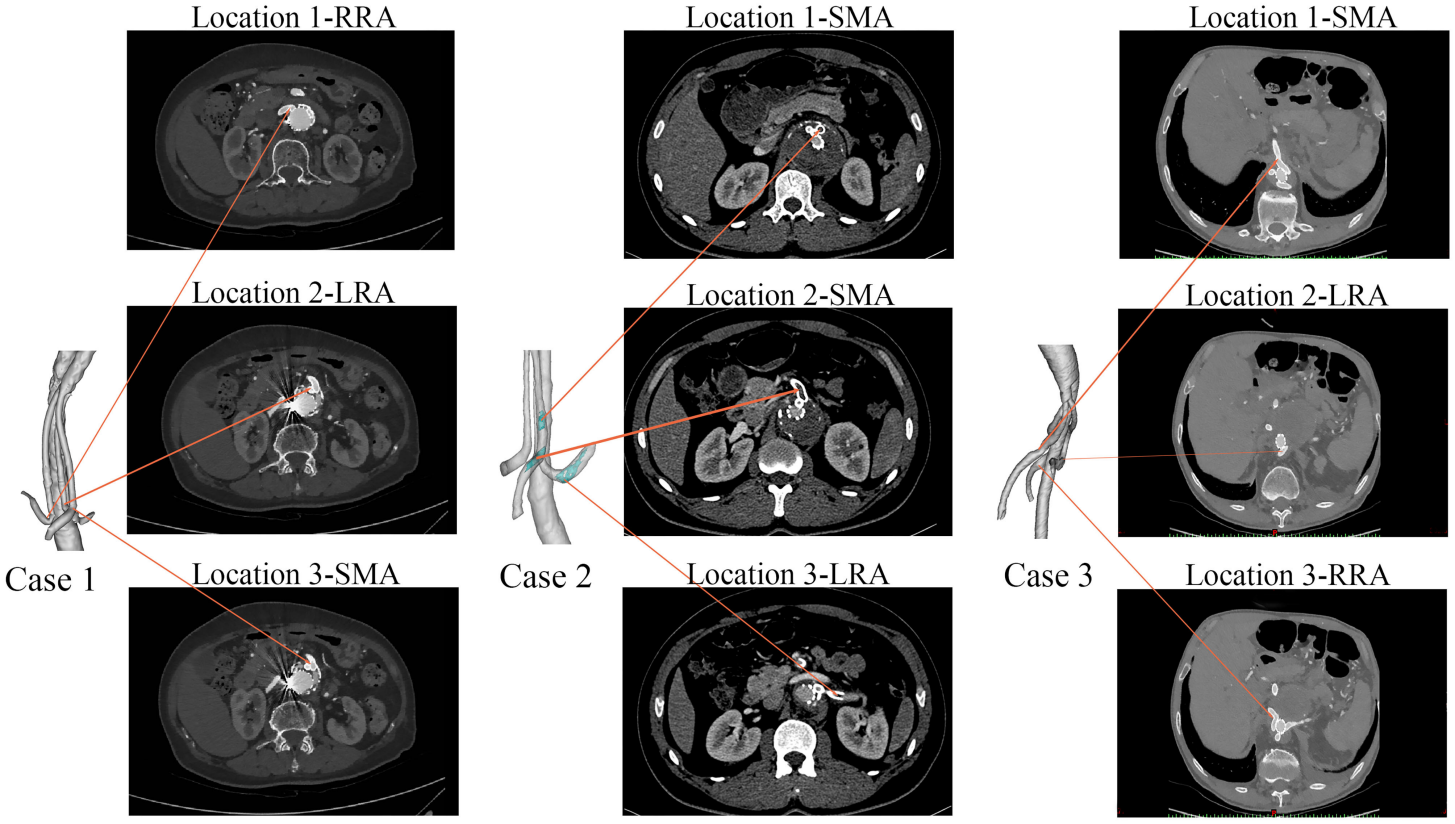
The follow-up CT scans of the three cases. Case 1 had no thrombi in the branches, Case 2 had branched stent-graft thrombosis (marked in green), and Case 3 had no thrombi in the branches.

The celiac artery (CA), superior mesenteric artery (SMA), left renal artery (LRA), and right renal artery (RRA) were repaired in Case 1; the SMA and the bilateral renal arteries were repaired in both Case 2 and Case 3. In order to unify the models for comparison between different cases, the celiac artery in the Case 1 model was removed. In the case 2, the adopted model was the one with actual thrombosis developed in the BSG, which was confirmed by the CT scan (Figure 3). The models were smoothed and optimized in Geomagic Studio software (3D System, Morrisville, NC, USA) for the meshing process.

### Mesh Generation and Computational Flow Dynamics (CFD) Simulations

Models were meshed using ICEM software (ANSYS, Inc., Canonsburg, PA, USA). In order to achieve the same degree of accuracy for all simulations, the same maximum global base cell size of 2 mm was used for each model. A hybrid meshing method (11) comprising both tetrahedral and hexahedral elements was used in all models. In addition, prism layers were created near the boundaries to ensure the accuracy of the model meshing. The total number of elements in models of case 1–3 is 993470, 805931, and 785617, respectively. The meshes of three models are shown in Figure 1B. The mesh sensitive analysis did not reveal any significant difference of the vessels among 3 cases. Furthermore, the blood flow of the visceral arteries was not obviously altered between the cases. There was no significant association observed for pre and post-operative anatomy. The hemodynamic analysis did not recognize any anomaly alteration of hemodynamic parameters at the first month and 6 months among cases.

### Boundary Conditions

The simulations were performed under pulsatile flow conditions, with the velocity profile (Figure 1C) as the inlet boundary conditions (12–15). *Outflow* conditions were used as outlet conditions, with the volume flow divided into 20.2% to the SMA, 19% to each renal artery, and 41.8% to the infrarenal aorta (16, 17). The flow ratio of each branch is marked in Figure 1B.

### Assumptions and Governing Equations

The blood was assumed to be an incompressible Newtonian fluid (18) with a dynamic viscosity of 0.0035 kg·m^−1^·s^−1^ and a density of 1,050 kg/m^3^. The numerical simulation was based on the Navier-Stokes equation (neglecting gravity) and the conservation of mass:

(1) $$\begin{array}{rcl} \rho \left[\frac{\partial \vec{u}}{\partial t}+\left(u\cdot \nabla \right)\vec{u}\right]+\nabla p-\mu \nabla^{2}\vec{u} & = & 0 \end{array}$$

(2) $$\begin{array}{rcl} \nabla \cdot \vec{u} & = & 0 \end{array}$$

where $\vec{u}$ and *p*, respectively, represent the fluid velocity vector and the pressure, while ρ and μ, respectively, are the density and viscosity of blood. The CFD software package ANSYS Fluent 14.5 (ANSYS, Lebanon, NH, USA) was used for the simulations. The pressure-based solver and SIMPLE algorithm were used for calculation.

The convergence criterion was set to 10^−5^ for both continuity and velocity residuals. A uniform time step of 0.005 s was chosen for all simulations. For each model, four cardiac cycles were carried out in each simulation process to obtain a periodic solution (19), and the results of the final cycle were used for post-processing and analysis (20).

### Wall Shear Stress (WSS)-Related Parameters

This study focused particularly on the various near-wall hemodynamic (NWH) parameters that have been shown to have an effect on thrombus formation. Therefore, the time-averaged wall shear stress (TAWSS), used to describe the general features of WSS, in the pulsatile cycle was analyzed. The TAWSS was defined as follows:

(3) $$\begin{array}{r} TAWSS=\frac{1}{T}\int_{0}^{T}|WSS\left(s,t\right)|\cdot dt \end{array}$$

where *T* is the duration of the cardiac cycle, *WSS* is the instantaneous wall shear stress vector, and *s* is the position on the vessel (or stent-graft) wall.

The oscillatory shear stress index (OSI) is a parameter that can describe the changing frequency of the WSS direction. It ranges from 0, where the flow is unidirectional and does not oscillate, to 0.5, where the WSS direction frequently changes.

The OSI on the inner wall of the models was calculated as follows (21, 22).

(4) $$\begin{array}{r} OSI=0.5\left[1-\left(\frac{\left|\frac{1}{T}\int_{0}^{T}WSS\left(s,t\right)\cdot dt\right|}{\frac{1}{T}\int_{0}^{T}|WSS\left(s,t\right)|\cdot dt}\right)\right] \end{array}$$

where *WSS* is a vector parameter whose direction changes with the cardiac cycle time, *T* is the duration of the cardiac cycle, and *s* is the position on the vessel (or stent-graft) wall.

The relative residence time (RRT) is generally used to characterize the length of time that particles stay near the wall (23). This metric can reflect both OSI and TAWSS (24). The RRT is defined as:

(5) $$\begin{array}{r} RRT=\frac{1}{\left(1-2\cdot OSI\right)\cdot TAWSS} \end{array}$$

### Data Analysis and Statistics

Qualitative and quantitative analyses of the full dataset were performed to observe local/global influences of the parameters of interest and their inter-correlation. Depending on the normality of the data, a 2-tailed *t* test or Mann-Whitney rank sum test was used to examine differences between continuous variables at the 4 time points (Replicated 3 times for each sections). The level of significance among the 4 hemodynamic parameters of interest (Velocity, TAWSS, OSI, RRT) were determinated using one-way analysis of variance (ANOVA) followed by Dunnett's multiple comparisons test. Statistical analyses were performed using R (R Foundation for Statistical Computing, Vienna, Austria; http://www.r-project.org) and GraphPad Prism® version 7 (GraphPad, San Diego, CA); statistical significance was defined as *p* < 0.05.

## Results

### Clinical Outcomes

Table 1 shows the demographic characteristics and clinical outcomes of the three patients. No complications were observed during the postoperative period in any of the three cases. During the follow-up period, Case 2 showed abdominal pain at 6 months after hospital discharge. CT angiography revealed that there was thrombosis in the BSG of the SMA and LRA (Figure 3). However, no IST was detected in either Case 1 or Case 3 (Figure 3).

**Table 1:** Demographic characteristics and clinical outcome of three cases.

	**Case 1**	**Case 2**	**Case 3**
Height (cm)	156	180	172
Weight (kg)	61	90	79
BMI	25.1	27.8	26.7
Gender	Female	Male	Male
Age	65	61	68
Follow-up time (months)	24	20	16
Blood pressure (mmHg)	130/91	120/81	150/90
BSG-IST	No	Yes	No
Velocity (m/s)	>0.6	<0.6	>0.6
TAWSS (Pa)	>0.8	<0.8	>0.8
OSI	<0.2	>0.2	<0.2
RRT (s)	<5	>5	<5
Stent-graft	Endurant^TM^, 36-14-120 mm; 6*150 mm, Viabahn^TM^	Endurant^TM^, 38-14-120 mm; 6*150 mm, Viabahn^TM^	Endurant^TM^, 30-14-120 mm; 6*150 mm, Viabahn^TM^

*BMI, body mass index; BSG, branched stent-graft; IST, in-stent thrombosis; TAWSS, time-averaged wall shear stress; OSI, oscillatory shear stress index; RRT, relative residence time*.

### Flow Velocity

**Figure 4:**
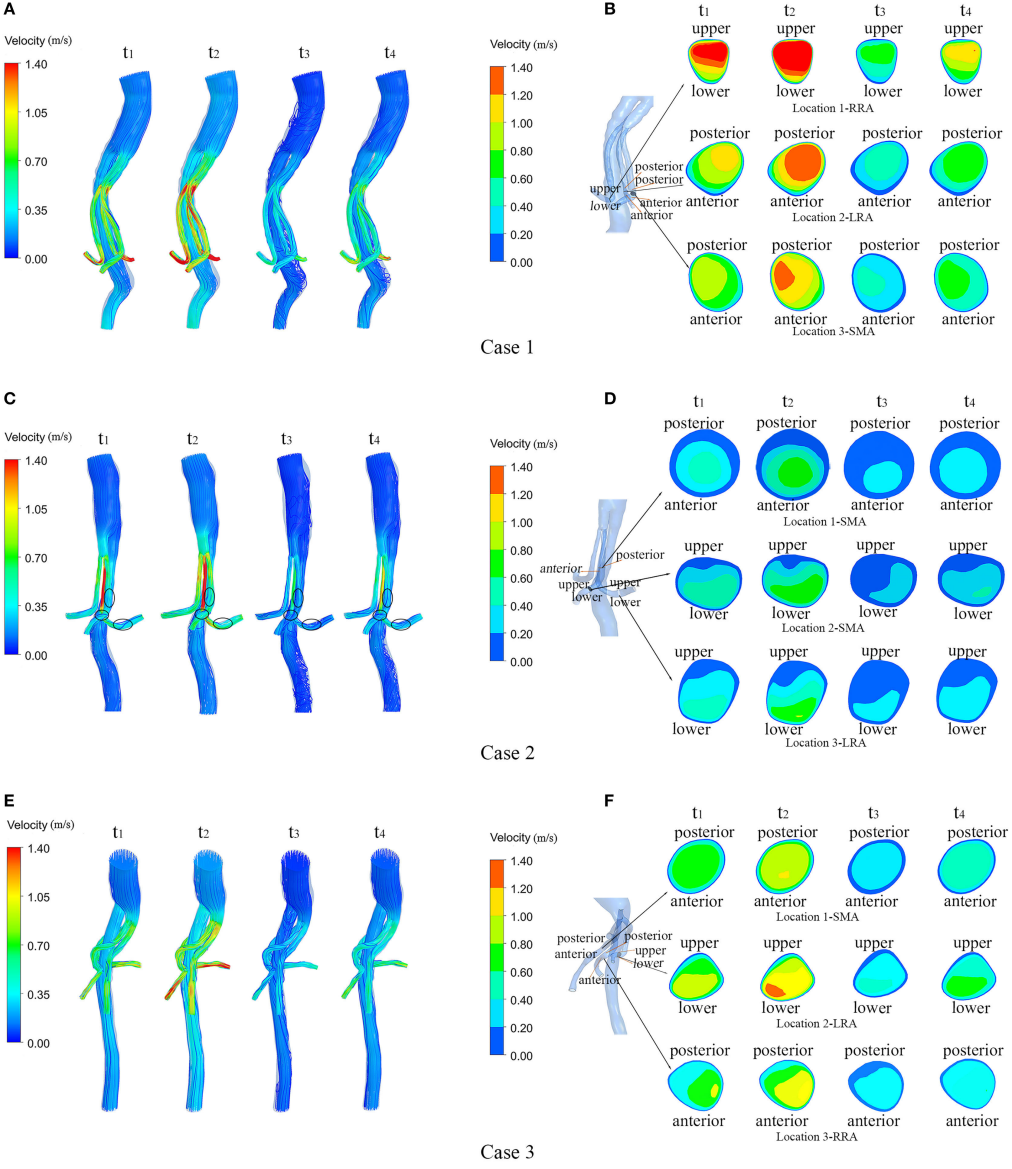
Velocity streamlines and continuous contours of velocity through a cardiac circle in each of the three cases. **(A,C,E)** show the velocity streamlines of the three cases at four typical moments (t1–t4) during a cardiac cycle; **(B,D,F)** show the locations of the sections in each model and the contours of flow velocity in the marked sections at four typical moments (t1–t4) during a cardiac cycle in the three cases.

Figure 4 shows the flow velocity in the three cases at four successive moments in the last cardiac cycle using four simulations (*t*_1_ = 0.125 s, *t*_2_ = 0.225 s, *t*_3_ = 0.435 s, *t*_4_ = 0.62 s). In order to reveal the flow characteristics of the BSG, the parameters are illustrated on the branched arterial region. The streamlines for the three cases are shown in Figures 4A–F, and the magnitude of the velocity is indicated by the color. The locations of thrombosis in Case 2 were precisely matched with the identified low-velocity areas in Figure 4C where IST occurred in the BSGs. In order to investigate the velocity change in these low-velocity regions, cross-sections were taken from the models of Case 1, Case 2, and Case 3. The velocity contours in different sections are also shown (Figures 4B,D,F). It was observed that the thrombosis/IST areas in Case 2, marked with black circles (Figure 4C), seemed to have lower velocity than the corresponding areas in Case 1 (Figure 4A) and Case 3 (Figure 4E) and lower velocity than the adjacent upstream and downstream areas in Case 2.

### WSS, TAWSS, OSI, and RRT

**Figure 5:**
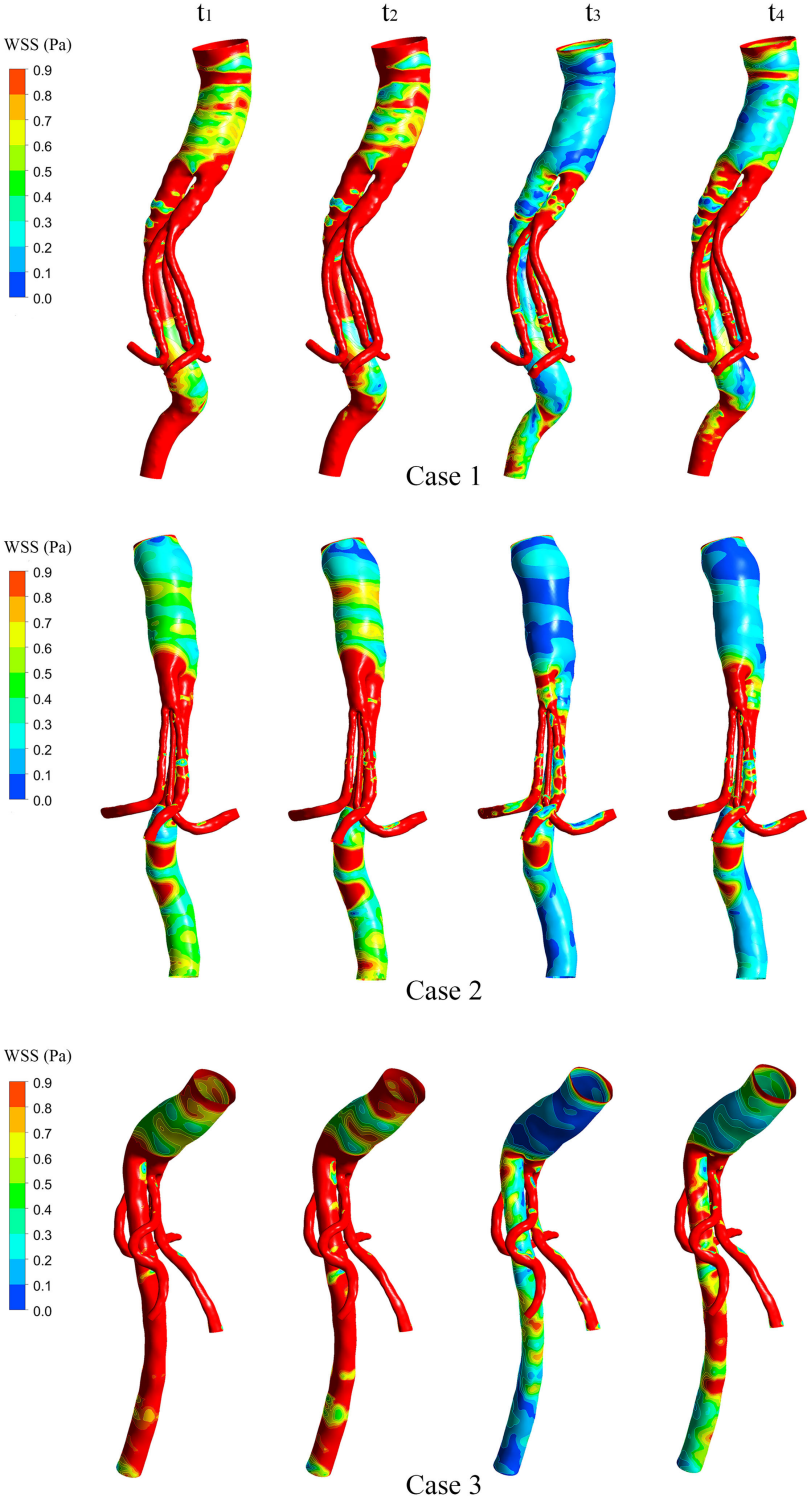
The wall shear stress (WSS) distributions of the stent-grafts in three cases. The WSS is presented at the same four selected moments (t_1_-t_4_) in each case.

**Figure 6:**
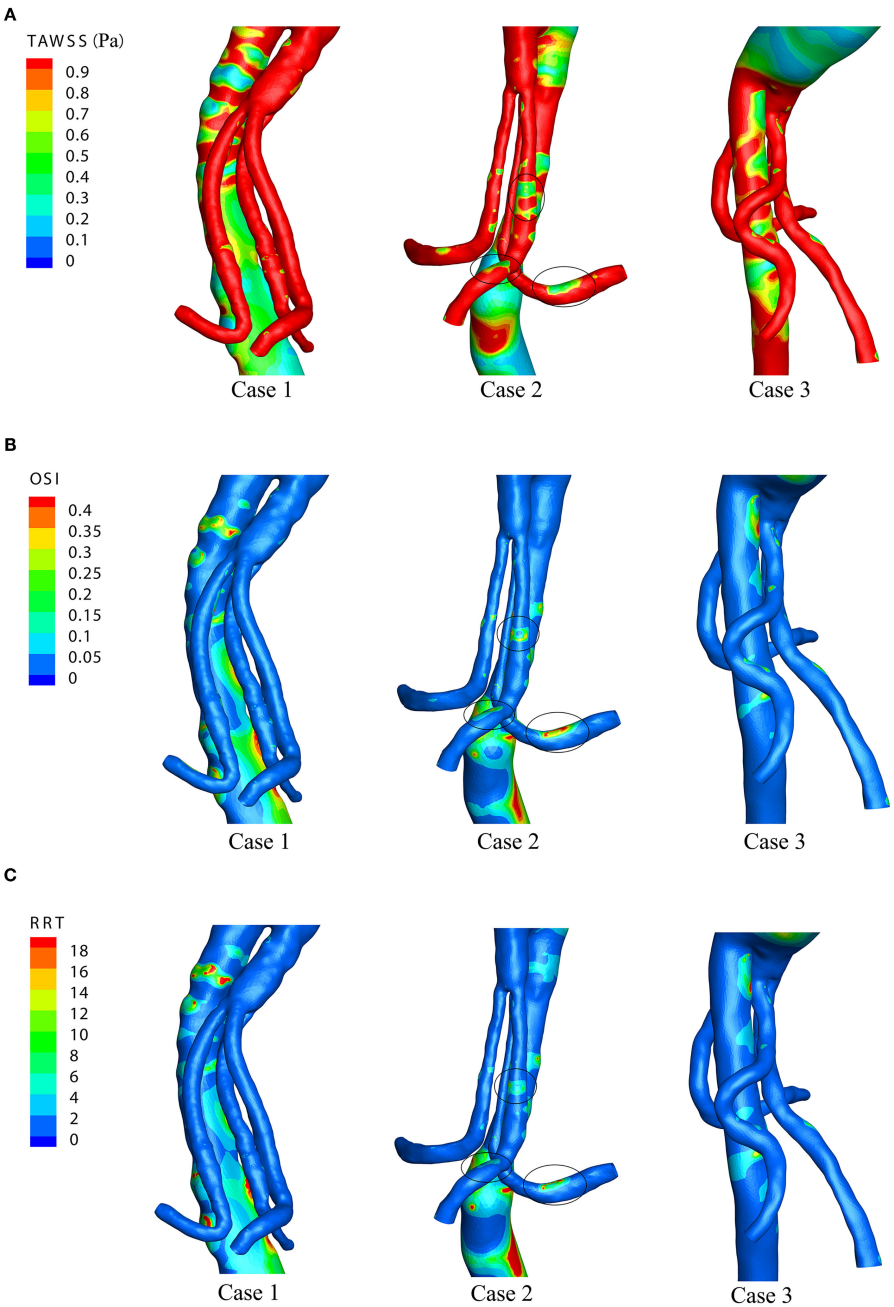
The time-averaged wall shear stress (TAWSS), oscillatory shear index (OSI), and relative residence time (RRT) distributions of the three cases. **(A)** TAWSS distributions. **(B)** OSI distributions. **(C)** RRT distributions.

Figure 5 shows the WSS distributions of the BSG through four successive moments in a cardiac cycle in each of the three cases. There was no remarkable change in the WSS along the BSG in Case 1 or Case 3. Nevertheless, low-WSS regions appeared over the course of the pulse cycle in Case 2, and the regions matched the IST area. Therefore, the hemodynamic parameters, including TAWSS, OSI and RRT, were analyzed to visualize the quantitative characteristics of the three cases. Contours of TAWSS, OSI and RRT on the walls of the stent-grafts are shown in Figures 6A–C. We found that the IST areas consistently matched the regions in which TAWSS, OSI and RRT were significantly altered in the BSGs. The IST area had a consistently lower values of velocity (<0.6 m/s) and TAWSS (<0.8 Pa), whereas a higher values of OSI (>0.2) and RRT(>5 s) than the non-thrombotic regions of the BSG.

## Discussion

Since PMSGs were introduced for the repair of complex aortic aneurysms (25), studies have amended the technique and demonstrated its feasibility and durability for patients with complex aortic aneurysms (26–28). Despite high technical success, however, visceral branch IST remains one of the leading complications after BSG implantation (29, 30). The flow characteristics of the BSG and the relationship between these characteristics and the IST remain largely unknown. Therefore, CFD with patient specific models were applied in this study to evaluate the hemodynamic mechanism of thrombosis within the visceral branches and explore IST-related hemodynamic parameters.

Low velocity is known to be associated with thrombosis. As shown in the velocity streamline diagram (Figure 4), the BSGs of Case 1 and Case 3 had higher velocities and more uniform flow patterns than the BSG of Case 2, and IST was observed in the marked areas (black circles). In Case 2, the velocity was significantly lower in the three marked (thrombosis) areas than in the upstream and downstream areas of the BSG (Figure 4) or in the corresponding locations of the other two cases.

Theoretically, low velocity induces a low TAWSS distribution (31). Our result demonstrated that the regions with low TAWSS (<0.8 Pa) (Figure 5) matched the regions with low flow velocity (<0.6 m/s) (Figure 4). Owing to the relevance of the WSS-related NWH hemodynamic parameters (Figure 6), we compared the TAWSS, OSI (an index to evaluate the fluctuation of the blood flow) and RRT and identified the consistent tendencies of these parameters. It has been reported that low WSS, high OSI and high RRT will promote platelet aggregation and ultimately lead to thrombus formation (31–33). Conti et al. showed that the femoro-popliteal in-stent thrombosis is characterized by larger diameter, low tortuosity, low flow velocity, low helicity, and low wall shear stress (34). Leg bending induces an overall increase of arterial tortuosity, helix flow, and reduces flow velocity which may furtherly promote the luminal area exposed to thrombosis related hemodynamic parameters. However, compared to the lower extremity, the stent-graft implanted in the visceral arteries were relatively steady to the lumbar vertebra, which may produce less helix flow rotation. Therefore, we used the established parameter-oscillatory shear index (19, 35) instead of helicity to address the turbulence in the endovascular aortic repair. In our results, the flow characteristics of TAWSS, OSI, and RRT were consistent in the non-thrombotic areas, whereas hemodynamic alteration appeared solely in the IST area. By analyzing the distribution of these parameters, we showed that the IST areas (Figure 3) matched the low-velocity areas (Figure 4). Furthermore, IST areas matched the areas of reduced TAWSS (Figure 6A), elevated OSI (Figure 6B), and RRT (Figure 6C). Our data have shown the hemodynamic parameters at the first month rather the 6 months. The configurations of the 3 patients with BSGs were not rigorously changed. The hemodynamic parameters were not statistically different at the first month and 6th months. The abnormal hemodynamics properties may not be observed at the very beginning. But it might be discovered and used to anticipate early thrombosis in a retrograde fashion. Together, these data demonstrated that hemodynamic perturbations introduced radical changes in hemodynamic parameters, which may predispose patients to IST. Alterations in hemodynamic parameters may be able to predict IST.

In a systematic review of off-the-shelf or physician-modified fenestrated and branched endografts, the author suggested that off-the-shelf and physician-modified technology seems effective and safe in both the elective and acute clinical outcome for the treatment of complex aortic aneurysms (29). The clinical outcomes of these techniques have been compared and reported in the articles (3, 36). Until for now, there is lack of literature that compared the hemodynamic differences under PMSGs, CMDs, or Off-the-shelf devices. With similarity in configurations, however, the hemodynamic environment underlying these modalities were unknown and it should be of great value to analyze the differences of hemodynamic characteristics. A variable flow properties characterized by flow separation, stagnation, low fluid velocity, and low WSS are associated with thrombosis and stenosis (10, 37). The curvature of the device and the change in diameter near the curved region can both contribute to these hemodynamic changes. In clinical practice, BSGs longer than the original vessels are almost invariably required for conveniently cannulation in the treatment of complex aortic aneurysms. Although a longer BSG would provide more gradual changes in momentum and allow blood flow to change gradually before reaching the target vessel (38), it would result in a curved portion, where the blood flow would be disturbed and thrombus-prone environments would be created. The hemodynamic parameters are known to be influenced by the highly curved and tortuous areas, which were commonly seen in the endovascular management for the complex aortic aneurysm. But what is not adequately described are, which certain types of hemodynamic changes (due to highly angled areas) were related to the in-stent thrombosis and whether these changes were of wide suitability among different techniques. In the present study, all three of the regions that developed IST after the procedure were at the bend of the BSG, which suggested that sharp curvature in a stent-graft adversely affects blood flow near the curved section, thereby accelerating thrombus formation. Therefore, we advocate preoperative planning to avoid sharp curvature at the bends of endografts.

Note that the PMSGs is a non-standardized technique and the long-term consequences of modifications remain unknown (39).

Research has illustrated the evolution from physician-modified to company-manufactured fenestrated-branched endografts in the treatment of complex and thoracoabdominal aortic aneurysms (36, 39). Through the evolution of devices, BSGs are typically used in multibranched endovascular aortic repairs. Despite the refinement of stent-grafts, preoperative design of the morphological configuration of the BSG should be considered. Our results suggested that sharp curvature in the BSG should be avoided and that early postoperative hemodynamic assessments of the BSG could assist in detecting predisposition toward IST after the operation. If the calculations identify regions with significantly changed hemodynamics in the BSG, special attention needs to be paid to these regions, as they may have an increased risk of IST. Our data indicated that relatively low velocity (<0.6 m/s) and TAWSS (<0.8 Pa) distributions and high OSI (>0.2) and RRT (>5 s) distributions were associated with the IST in the BSG. Therefore, high OSI and RRT and low TAWSS may predict the areas where IST will develop.

In contrast to the previous ideal model used to study branched stent configurations (16, 38), the models used in this study were derived from clinical cases, augmenting current knowledge with “real-world” data. Moreover, the models in this study provided important insights into the velocity and WSS distributions of BSGs.

## Limitation

This study has several limitations that should be mentioned. First, only the fluid domain of the blood vessel was used for simulation. Although the interaction between stent-grafts and blood flow with the pulsatility of the flow were not considered in this study, the results are still reliable because it was a common practice in CFD analysis for fenestration endograft (40). Second, the stiffness and conformability of the bridging stents are important and it should be addressed by rigorous mechanics analysis. However, this condition cannot be achieved at present. Third, it should be noticed that the PMSGs was used outside the instruction for use and it should be used in the condition when validated treatments are not available. PMSGs is a non-standardized technique and the long-term consequences of modifications remain unknown. Although the patient specific models may improve computational simulation and integrate further details, the sample size was limited in our study. We recommend further evaluation with a large sample size, patient-specific boundaries, and long-term follow-up to validate these findings.

## Conclusion

This study found that anomaly hemodynamic parameters predispose patients to IST within BSGs. Analysis of these parameters in the early postoperative period may be beneficial for identifying and remediating IST after multibranched endovascular aortic repair.

## Data Availability Statement

The data that support the findings of this study are available from Peking University People's Hospital, but restrictions apply to the availability of these data, which were used under license for the current study and therefore are not publicly available. The data are, however, available from the authors upon reasonable request and with permission of the Ethical Review Board, Peking University People's Hospital.

## Ethics Statement

The authors confirmed that all experimental protocols involving human data in this study were in compliance with national/international/institutional guidelines or the Declaration of Helsinki. Informed consent was obtained from all the patients, and the study protocols were approved by the Ethical Review Board and Statistics Department of Peking University People's Hospital (No. 2017 PHB166-01). Consent for publication was obtained from all participants.

## Author Contributions

M-YL and WL were contributed to the conception and study design, obtaining funding, writing the article, and critical revision of the article. YJ and JL were contributed to the data collection, analysis, and interpretation. SZ was contributed to the obtaining funding.

## Conflict of Interest

The authors declare that the research was conducted in the absence of any commercial or financial relationships that could be construed as a potential conflict of interest.

## Abbreviations

AAA: abdominal aortic aneurysm
EVAR: endovascular aortic repair
BSG: branched stent-graft
IST: in-stent thrombosis
CT: computed tomography
CA: celiac artery
SMA: superior mesenteric artery
LRA: left renal artery
RRA: right renal artery
TAWSS: time-averaged wall shear stress
OSI: oscillatory shear stress index
RRT: relative residence time
PMSGs: physician modified stent grafts
CMDs: custom made devices.
